# Supplementary material for: Are carbon emissions trading and green financial instruments synergistic? -Comprehensive quantitative research based on content analysis
Source: PLoS One. 2024 Mar 7;19(3):e0298601. doi: 10.1371/journal.pone.0298601 (PMC10919601; doi:10.1371/journal.pone.0298601)
Supplement: S1 Data — (ZIP) [file pone.0298601.s001.zip › Data/地级市Z_F工作报告.zip!/░▓╗╒/░÷▓║╩╨/└┤╫╘╣1⁄2╓┌║┼í╛┬φ┐╦╩2╛▌═°í┐_2007.docx]

各位代表：

    现在，我代表市人民政府向大会报告工作，请予审议，并请市政协委员和其他列席人员提出意见。

    一、2006年工作回顾

    刚刚过去的2006年，是“十一五”规划开局之年。在中共蚌埠市委的坚强领导下，全市上下以科学发展观为统领，认真贯彻“推进跨越式发展、实现争先崛起”的总体要求，求真务实，开拓进取，完成了市十三届人大四次会议提出的目标任务，经济和社会发展取得了新的成就。

    ——推动经济较快发展，主要目标全部实现。预计全年实现地区生产总值350亿元，同比增长12%。一、二、三次产业增加值分别增长8.7%、14.5%和11.5%，其中工业增长14.6%。全部财政收入45.2亿元，增长14.5%，其中地方财政收入16亿元，增长24.7%。全社会固定资产投资完成144亿元，增长15.4%。出口总额2.43亿美元，增长11%。实际利用外资1.42亿美元，增长22.4%，引进市外资金77亿元，增长40%。社会消费品零售总额133亿元，增长14.2%。城镇居民人均可支配收入10150元，农民人均纯收入3150元，分别增长14.4%和13.3%。物价保持平稳，居民消费价格涨幅控制在3%以内。单位生产总值能耗下降4%左右。

    ——落实工业强市战略，工业发展步伐加快。紧紧抓住发展工业这根经济工作主线，积极实施“8765”工业振兴工程，制定了优先扶持工业发展的43项措施，促进政策向工业倾斜、力量向工业集中、资源向工业集聚，初步形成了决战工业之势。大力培育有竞争力的产业，编制完成了部分重点产业发展规划，其中生物质产业和精细化工产业已被纳入省级重点产业规划体系。大力培育优势企业，全年新增规模以上工业企业49家。丰原集团与中粮集团达成战略性合作协议。天宝集团建成“498”发动机等项目，当年实现产值22亿多元。蚌埠卷烟厂完成新的技改项目，年生产能力达到80万箱。八一化工扩大与外企的合作，一批新项目开工建设。宏业集团向纺织、玻璃领域拓展，企业规模壮大。环球药业、华益公司等企业扩建项目建成投产。金黄山公司、浙泰公司、通达公司、皖酒集团等企业发展加快。国有企业改革继续推进，上市公司股权分置改革全部完成。千方百计盘活“休克企业”，促成花园油脂、麦科特纺织、天兔纺织、盛通玻璃等企业恢复生产，实现华光鲲鹏玻璃厂、顺达汽车改装厂等与优势企业嫁接。组织银企对接活动，一批中小企业获得信贷支持。品牌战略取得进展，丰原集团BBCA牌饲料级赖氨酸获得中国名牌产品称号。推进组建了滤清器、化工、纺织服装等行业协会。争取老工业基地城市政策取得重要进展。6个园区通过国家审核并全部定为省级园区。2006年全市工业增加值超过115亿元，其中规模以上工业实现增加值86亿元、利税32.8亿元，分别增长19%和12.6%。

    ——积极推进新农村建设，“三农”工作取得新的进展。当年投入财政资金7045万元，加强农业综合开发。推广良种良法，落实粮食最低收购价政策，粮食总产量达245万吨，总产和单产均创历史最好水平。大力发展养殖业，建成标准化养殖小区24个。加大动物疫病防控，全年无重大动物疫情发生。加快农业产业化步伐，新增省级龙头企业6家、省名牌农产品9个。农民专业合作经济组织发展到227个，带动农户21.7万户。培训农村劳动力1.4万人，全市劳动力转移55万人，劳务收入达到25亿元。认真兑现“三项补贴”政策，增加了农民现金收入。规范涉农收费，进一步减轻了农民负担。推进林权制度改革试点，新增林权登记面积1.3万亩。实施新农村建设“111”试点工程，编制完成44个乡镇的总体规划。加快农村基础设施建设，搬迁改造“空心村”8个，完成移民建房2350户，建成农村公路1013公里，建设农田水利工程3021处，完成14个农村安全饮水工程。县域经济发展加快，工业总产值、固定资产投资、财政收入等重要指标均有较大幅度增长。

    ——完善商贸基础设施，服务业保持较快发展。继续推进商业零售网点体系建设，创新城乡流通方式，实现了城乡市场消费同步增长。“万村千乡”市场工程建设初见成效，新建455个农村日用品消费店和农资连锁店，农村消费环境得到改善。推进社区“双进工程”，积极开展创建商业示范社区活动。国美电器蚌埠店、家乐福蚌埠店已经建成，经济开发区商业步行街主体工程完工，营造了商业发展新优势。住房消费活跃，全年成交额17.3亿元。汽车、通讯、旅游等消费热点加快形成。批发和零售贸易、住宿餐饮等传统产业对经济增长的拉动作用增强。银行、证券、保险业发展较快，农村信用社改革取得突破，4家农信联社全部挂牌。对外贸易平稳增长，我市被授予“国家科技兴贸出口创新基地”称号，金威公司、昊方公司被授予“国家汽车零部件出口基地企业”称号。整顿规范市场秩序、打击非法传销等行为取得阶段性成效。

    ——招商引资不断升温，重点项目进展顺利。加大驻点招商力度，全市派出109个驻外招商小组，在重点区域设立了8个招商代表处，从长三角地区引入的资金占全市引资的60%以上。强化县区和开发区招商责任，高新区、经济开发区引资超过15亿元，三县引资均超过7亿元，龙子湖、蚌山和淮上区超过5亿元。精心组织招商活动，成功举办了2006中国花鼓灯歌舞节暨蚌埠投资贸易洽谈会，签约47个项目，总投资近70亿元。加强项目跟踪服务，提高了签约项目履约率，87个5000万元以上项目顺利落户。调动企业招商引资积极性，蚌埠玻璃设计院、市建投公司、昊方公司等依托技术、产品等优势引进合作项目，取得了明显成绩。高度重视重大项目谋划工作，组织编制了千亿元规模的“十一五”项目储备库，其中建立了420亿元的工业投资项目库，52个工业项目列入省技改“百千工程”。加强领导和调度，推进重点项目建设，全年开工35个项目，竣工24个项目。交通投资完成4.25亿元，蚌埠新港一期投入使用。水利投资完成6.6亿元，荆山湖进退洪闸、荆家沟站涵、天河封闭堤等工程竣工验收。110千伏蚌埠中心输变电、固镇连城变改造、怀远新城输变电等项目竣工。京沪铁路蚌埠段电气化改造完成。投资结构进一步优化，完成工业投资57亿元，占城镇固定资产投资的44%，工业成为投资的重点领域。

    ——城市面貌继续改善，城市管理有所加强。编制完成了市区2005—2010年建设规划，启动土地利用总体规划修编工作，基本完成城市道路、排水工程专项规划。加快城市道路建设，延安路和航华路南延段竣工通车，胜利路西段整修完毕，39条三、四类道路改造完成，涂山路西段改造全面启动。实施东海大道景观整治，开工建设龙子湖桥头公园。推进城市美化亮化，对主干道两侧11个重要节点实施亮化，完成了50条街巷路灯改造，火车站广场改造一新。新建了友谊广场、大禹文化广场、城市防洪景观绿地，增加城市绿地2154.5亩。南山儿童公园经改造后免费开放。加快治污工程建设，市第三污水处理厂开工，新建和改造垃圾中转站（收集点）21个、公厕23座，垃圾填埋场部分投入使用。11个城中村改造启动。全年城市基础设施建设完成投资10亿元左右。保持房地产健康发展，竣工面积100万平方米，建成政策性保障住房26.3万平方米。开展市容环境综合整治试点，集中整治影响市容的突出问题，清理城市“牛皮癣”和非法广告，城区秩序有所改观。打击非法买卖土地，拆除违法建筑6.8万平方米。加大征地拆迁工作力度，多年遗留的21个项目拆迁进度加快，拆迁秩序好转。划定城市生活饮用水水源保护区，削减水污染物排放量，严格监督工业企业达标排放情况，重视大气和噪音污染防治，环境保护和污染治理工作得到加强。

    ——加快发展社会事业，和谐蚌埠建设取得新进展。全年用于科教文卫等方面的经费净增7100万元。出台了鼓励科技创新的新政策，怀远被列为科技部科技富民强县试点县。“两基”教育通过国家检查，高考创历史最好成绩，蚌埠学院“去筹升本”通过教育部高校设置专家组评估。加强历史文化遗产保护，花鼓灯、泗州戏被列入首批国家非物质文化遗产保护名录，长淮卫村、顺河街等被评为安徽省历史文化名村、街区。龙子湖风景区、张公山公园等被评为国家3A级旅游景区，丰原集团、禾泉农庄被评为全国工业、农业旅游示范点。市医疗救治中心建成，市紧急救援中心主体完工，三县疾控中心、传染病区投入使用。我市运动员在省十一届运动会上取得金牌数、奖牌总数和团体总分第二的历史最好成绩。新一代天气雷达、气象科普公园主体完工。广播电视发射塔开工建设。市体育场维修改造启动，新体育馆设计已经完成。实施积极的就业政策，新增就业岗位3.8万个，帮助2.8万名下岗失业人员实现再就业。以扶持创业带动再就业，全年发放小额担保贷款3904万元，减免税费1540万元，惠及5100余人。城镇登记失业率控制在4.5%以内。完善社会保障体系，扩大社会保险覆盖面，城镇职工“五险”参保人数增加，社会保险基金收支基本保持平衡。高度重视特殊群体和困难群众基本生活保障，提高了最低工资标准和企业离退休人员基本养老金发放标准，发放城市居民最低生活保障金6107万元，对3.7万农村困难群众给予生活救助。怀远县新型农村合作医疗试点工作取得成效。城市社区卫生服务覆盖率提高到87%。

    努力维护社会稳定。加强突发公共事件应对工作，组织人民防空、重大食品安全、群体性事件、反恐等应急演练。认真解决信访积案，整治和规范信访秩序，赴省上访批次和人次大幅度下降。强化社会治安综合治理，开展打黑除恶专项行动，刑事发案率有所下降。加强安全生产监督管理，全年未发生重特大安全事故，连续八年获得省安全生产先进市称号。大力整顿食品药品市场，保障群众饮食用药安全。深入实施关爱女孩行动，出生人口性别比下降3.82个比值。双拥共建、国防动员、文明创建、民族宗教、社会科学、统计、人防、外事、档案、气象、地震和地方志等工作取得新成绩。

    ——推进机关效能建设，发展环境得到优化。以深化行政审批制度改革为突破口，全面加强机关效能建设，通过取消、下放、合并等方式，使审批项目由498项减少到298项，并全部纳入市行政服务中心集中管理，审批效率明显提高。进一步规范了行政事业性收费。加快优化创业环境，完善创业政策措施，全民创业取得较大进展，2006年新设立11个创业基地，新增私营企业1277家、注册资本金9.7亿元，新登记个体工商户8829户。主动接受市人大及其常委会的监督，充分采纳政协委员和社会各界人士建议，政府重要决策邀请市人大、政协领导参与，重大问题向市四大班子联席会议通报，科学民主决策水平提高。办理人大代表议案6件、建议135件，办理政协提案402件、建议案1件。加强与民主党派、工商联和人民团体的联系。利用电台、电视台和网络开通政风行风热线、政风行风面对面、市民心声等栏目，加强与市民联系沟通，妥善解决涉及群众切身利益的问题。贯彻落实《公务员法》，推进依法行政，强化监察和审计工作，查处了一批损害群众利益的突出问题和违法违纪案件。重视中长期发展战略研究，编制完成了“十一五”规划。完善目标管理和政务督查，促进了各项工作的落实。

    各位代表！过去的一年，我们在新的起点上迈出了新的步伐，成绩来之不易。这是中共蚌埠市委正确领导的结果，这是全市人民共同努力的结果。在此，我谨代表市人民政府，向全市各族人民，向广大工人、农民、知识分子、干部、驻蚌解放军指战员、武警官兵和政法干警，向国家和省驻蚌单位，向各民主党派、工商联、各人民团体和其他各界人士，向人大代表、政协委员，向离退休老同志，向在蚌埠创业的境内外投资者和建设者，向所有关心、支持和参与蚌埠现代化建设的海内外朋友，表示崇高敬意和衷心感谢！

    回顾过去，我们也清醒地看到，当前发展中还存在一些突出矛盾和问题。发展速度还不够快，与实现赶超先进地区的要求，与跨越式发展的要求，还有差距。经济结构还不够优，农业产业化进程不快，工业主导作用还没有发挥到位，骨干企业数量较少。经济增长方式还比较粗放，经济效益不高，加快发展与节约资源、保护环境的矛盾比较突出。发展后劲和活力不够强，带动全市发展的重大工业项目不多。城市建设和管理还不到位，不文明行为和市区脏乱差现象依然存在。政府职能转变还不到位，行政审批效率和为企业服务水平还需要继续提高。影响社会和谐的因素还有不少，一些关系群众切身利益的问题还没有得到很好解决。对于上述矛盾和问题，我们将继续努力，切实加以解决。

    二、2007年工作目标和要求

    2007年是本届政府任期最后一年，也是实施“十一五”规划的重要一年。做好今年工作，对于实现蚌埠争先崛起，完成全面建设小康社会起步阶段的目标任务，具有重要意义。综观今年经济发展形势，尽管我们面临诸多压力和挑战，但是总体上说仍然是一个机遇与挑战并存、机遇大于挑战的环境，一个有利于我们实现跨越式发展的环境。从外部环境看，世界经济可望继续保持增长态势，我国经济也正处于新一轮增长周期；东部地区和境外产业向我国中西部转移步伐加快；国家促进中部地区崛起、支持新农村建设的政策和我省促进“两淮一蚌”沿淮城市群发展的政策，逐步进入实质性操作阶段。从我们自身发展看，全市已经形成较好的发展基础和浓厚的发展氛围，发展思路更加清晰，发展信心更加坚定，长期积蓄的发展能量正在加速释放。面对十分难得而又可以大有作为的历史机遇，我们必须凝心聚力，乘势而上，因势利导，克服困难，加快开创蚌埠经济和社会发展的新局面。

    今年经济和社会发展工作的总体要求是：以邓小平理论和“三个代表”重要思想为指导，全面落实科学发展观，继续推进工业化、城镇化和农业现代化，继续抓住全民创业、招商引资、扩大投资不放松，继续深化改革和鼓励创新，继续促进经济结构调整和经济增长方式转变，继续重视和谐社会建设和解决民生问题，抢抓机遇，争先崛起，努力实现国民经济又好又快发展和社会和谐稳定。

今年经济社会发展的主要预期目标是：地区生产总值增长12%以上，其中一产增长5%，二产增长16%以上，全部工业增长16%以上，三产增长12%；财政总收入增长10.5%，其中地方财政收入增长11.9%；全社会固定资产投资增长20%以上，总量达到175亿元；招商引资总额达到100亿元以上，其中利用境外资金增长15%以上，引进市外资金增长20%以上；社会消费品零售总额增长12%；进出口总额增长11%；城镇居民人均可支配收入和农民人均纯收入均增长7%；城镇登记失业率、人口自然增长率控制在省下达的目标以内；单位生产总值能耗降低4%。

    上述目标安排，是在综合分析宏观经济运行环境和自身实际的基础上确定的，力求体现又好又快发展和实现跨越式发展的总体要求。发展是我们全部工作的主题，要完成本届政府任期目标，完成“十一五”规划确定的目标，应对省内各市竞相发展的竞争局面，增强全市综合实力，加快改善人民生活，促进和谐社会建设，我们就必须争取发展得更快。近年来我市经济处于稳步上升期，2006年人均生产总值已经达到1万元，这标志着经济发展进入一个新的阶段，今年我们将有一批工业项目投产达产，有多个重大基础设施建设项目开工或竣工，全市上下加快发展的积极性和热情不断高涨，我们具备发展得更好更快的有利条件。我们相信，只要全市上下齐心协力，埋头苦干，上述目标是能够实现的，在实际工作中我们还要力争能高则高、能快则快，争取发展得更好一些。

    三、2007年主要工作任务

    为了全面实现今年目标任务，我们必须重点抓好以下工作：

    （一）继续实施工业强市战略，促进工业提速增效

    没有工业的加快发展，就难以实现整个经济的加快发展。我们要进一步强化工业强市理念，用更多的精力谋划工业、抓好工业。

    积极培育重点工业产业。进一步做大以生物质和精细化工为主导的化工产业，建成丰原生化年产300吨秸秆酒精、2万吨玉米油，天润公司2万吨聚丙烯酰胺，八一化工1万吨对氨基苯酚、6万吨硝基氯苯等项目。培育壮大汽车零部件产业，建成天宝集团年产3000副中小型模具，昊方公司300万套汽车空调电磁离合器，金威公司100万套柴油水分离沉淀器总成等项目，开工建设液力公司年产27万米油缸和4万台变矩器项目。提升机械装备制造业水平，加快实施晶菱公司数控机床、数控铣床的技术改造和安瑞科350台天然气压缩机项目，开工建设振冲安利大中吨位汽车起重机项目等，促进安瑞科、华隆等企业搬迁。增强玻璃及玻璃深加工产业竞争力，加快凯盛玻璃新材料产业园首期项目建设，完成盛世公司年产800吨超宽幅功能光学玻璃、鑫民公司60平米燃煤窑炉节能改造项目。

    积极培育高新技术产业，扶持优势传统产业。支持新材料、新能源和电子信息产业发展，争取华皖碳纤维、新奥二甲醚一期投产，普乐太阳能电池一期、亿多电子项目建成，鼓励驻蚌科研院所在蚌建立高新技术产品研发和生产基地。促进烟草、纺织、食品、酿酒等传统产业发展，实施烟厂膨胀车间等3个改造项目，加快建设宏业肉类加工及出口基地、创业纺织30万锭棉纺一期等工程，推进康地万达肉鸡综合加工等项目。

    积极培育骨干工业企业。重点培育和打造具有较强竞争力和综合实力的企业集团，延长产业链，使其成为带动产业升级和集聚的主导力量。落实跟踪指导和服务措施，力争丰原集团、蚌埠烟厂、天宝集团、八一化工等15家行业骨干企业总产值达到200亿元以上。推进企业技术创新，引导企业建立和完善技术中心，不断提升自主研发能力，重点支持13家省级技术中心建设，培育具有自主知识产权和较强竞争力的创新型企业。实施品牌战略，争创中国名牌产品1个，省级名牌产品、著名商标9个。

    切实抓好工业园区建设。做好开发区扩区工作，加快园区基础设施建设。高新区抓紧申报国家级开发区。建立征地资金预存制度，保障工业投资项目用地，年内新增建设用地计划指标75%以上用于工业项目和工业园区发展。倡导集约用地，建设一批多层标准化厂房。有序推进企业“退市进园”。鼓励孝仪、龙亢、长淮卫、沫河口、秦集等有条件的重点乡镇建设工业集中区。

    全面落实加快工业发展的调控措施。制定鼓励发展的产业指导目录，完善市、县（区）两级工业企业服务与监测体系，密切关注重要生产资料和能源、运输等方面的供求变化，搞好衔接与协调，及时帮助企业解决困难。组建机械汽车、建筑材料行业协会，加强产业协作，推动本地企业产品配套。建立中小企业发展基金，推进政银企合作，加大对中小企业发展的资金扶持力度。积极开展资源综合利用工作，加快节能新技术、新工艺、新设备的推广应用，推进清洁生产，发展循环经济，对年耗能千吨以上标准煤的企业要进行重点管理和监督。

    （二）稳步推进新农村建设，加快振兴县域经济

    大力发展现代农业，加快培育农村新经济。继续推动农业产业化，扶持农产品加工龙头企业的发展，今年要新增市级重点龙头企业38家。抓好种植业5个产业带建设，新增标准化养殖小区20个。培育50个“一村一品”特色示范村。积极创建农产品品牌，力争新创名优产品10个。加快发展专业合作组织，力争2—3个进入省级试点。抓好3项农业科技创新项目，重点推广13项实用技术。

大力促进农民增收，支持农民创造新生活。继续发展劳务经济，提高劳务输出的规模和层次。鼓励和支持农民创业，建立农民创业贷款担保中心，拓宽创业农民的融资渠道。选择有条件的地区成立返乡创业者协会，提供相关创业服务。落实农业补贴政策，加强监督管理，增加农民收入，减轻农民负担。加快新农村建设试点镇基础设施建设，完成33个试点村规划，改造15个试点村。抓好内河圩堤除险加固，推进农田水利综合治理。新建农村道路800公里，桥梁10座，公路客运站7个。整体推进“十镇百村”水环境整治工程。

    深化农村各项改革，创新农村工作新机制。推进乡镇机构、农村义务教育和县乡财政管理体制等改革。开展农村社区建设试点。制定农村“一事一议”筹资筹劳管理办法，尊重农民意愿，发动农民参与村内基础设施建设。出台新的集体土地征地补偿标准，鼓励村组利用土地补偿款发展集体经济。深化农村信用社改革，力争组建1家农村合作银行，改善农村金融环境。加强村级自治组织建设和民主监督管理，做好第七届村委会换届选举工作。

    提高农民综合素质，积极培育新农民。加强农村社会主义精神文明建设，继续开展文明村镇、文明家庭创建，引导农民崇尚科学、移风易俗，逐步形成健康文明的农村新风貌。继续开展“三下乡”活动，提高农民文化、科技、法律等知识水平。整合各种职业教育和培训资源，开展技术培训，争取让5万以上农民通过培训掌握1－2门实用技术，提高农民创业致富本领。

促进传统经济向现代经济转变，实现县域经济发展的新跨越。要发挥好各县的特色和优势，依托县城和工业园区，加大招商引资力度，放手发展民营经济，加快发展县域工业，带动整个县域经济发展，为全市发展作出新贡献。

    （三）适应消费需求增长趋势，积极培育新型服务业

    继续抓好重点商业项目建设。建成沃尔玛购物中心和苏果超市，启动市卫校原址地块商业项目开发。支持市区大型零售市场、连锁超市和购物中心发展，提升商业层次。建成路桥日用品商城、通城国贸广场一期工程，形成淮上商贸区雏形。培育新型消费方式和新的消费热点，加快宝龙城市广场建设，在经济开发区引资新建一座高星级酒店。鼓励大型流通企业进入社区。推进农村市场建设，继续实施“万村千乡”市场工程，扩大农村消费。打击商业欺诈、非法传销，整顿和规范市场秩序。

    积极培育新型服务业。充分发挥蚌埠作为区域中心城市的优势，加快把有特色的新型服务业培育成新的经济增长点。依托交通枢纽地位，发展现代物流产业。规划建设姜桥物流园、丰原铁路专用线配套货场，发挥蚌埠新港、现代物流园的作用，筹建蚌埠新港二期工程；积极引进徽商集团等企业，建设钢材等专业市场，推进生猪联合交易市场建设。依托历史文化和自然山水等资源，大力培育文化旅游业。适应城市居民休闲需求，开发固镇园艺二场、新马桥干校观光梨园、沱湖水上游乐、怀远石榴采摘等一批城市近郊休闲观光和农家乐旅游项目；抓好景区、景点开发，启动锥子山景区、黑虎山－天河景区建设，推进垓下古战场、荆山－涂山景区、东－西芦山景区、大巩山－樵子涧景区、蚌埠闸水利风景区等项目的建设；利用周边地区旅游资源，加强互利互惠合作，努力将蚌埠建设成为皖北地区旅游集散地。积极培育会展、金融、证券、期货和信息咨询等服务业。

    （四）进一步加强项目谋划，狠抓重大项目实施

    加强项目谋划工作。增加投入、招商引资的前提和基础是必须有一批又一批好的项目。今年要根据自身优势、市场需求和经济社会发展需要，再研究提出一批新项目，要着重谋划一批投资上10亿元的有牵动性的重大项目，力争新增项目储备规模200亿元以上。要重点谋划好汽车工业园、船舶工业园、滤清器产业园、粮食产业园、电子产业基地等项目，做好合肥—蚌埠铁路客运专线、大庆路淮河公路桥、国家大型商品粮基地二期等项目前期工作。设立专项资金，对重大项目前期工作给予补贴或奖励，激发各方面谋项目、谋好项目的积极性。

    突出抓好重大项目建设。对续建的项目要加强调度，搞好协调，实行全方位服务，确保建设进度，重点抓好市政府直接调度的重大项目建设。要建成海螺水泥粉磨站、500千伏蚌埠输变电、淮河流域气象中心等一批项目，加快淮北大堤加固等项目建设进度。加快前期工作，尽快开工建设国电蚌埠发电厂、丰原铁路专用线、淮河复线船闸、平原洼地治理、洪泽湖抬高蓄水位影响处理工程等13个亿元以上项目。做好京沪高铁蚌埠段、蚌淮、濉明高速公路蚌埠段等国家、省重大项目的衔接和建设环境保障工作。激活民间投资，积极鼓励从资本市场融资、通过股权融资等，多渠道筹措建设资金。继续把工业作为投资重点，争取完成工业投资占城镇固定资产投资的50%。进一步严格责任制，落实项目建设主体的责任和部门跟踪服务制，强化调度和考核，做到奖惩到位；对重大项目实行市级领导“一对一”的联系制度，加强服务和协调，保障项目顺利推进。

    （五）强化招商引资工作，提高对外开放水平

    继续促使招商引资工作升温。推动县区招商，充分调动县区招商积极性和主动性，各县、区和高新区、经济开发区在增加招商引资数量的同时，要努力提高招商引资质量。推动企业招商，充分发挥骨干企业招商主体作用，利用特色资源、科研成果等优势，力争引进1—3家实力较强的战略投资者。推进驻点招商，依托现有节点，整合招商队伍，切实提高驻点招商实效。推进产业招商，围绕产业基地和重点产业，努力引进配套企业，延长产业链，促进产业集聚。推进会展招商，有针对性地开展投资推介和贸易洽谈，提高重大招商活动成效。改进招商服务，施行重点招商项目“绿色通道”制度，推动一批重大项目落户，提高项目履约率和开工率。继续加大利用国外贷款力度，重点推进城市环境综合治理利用世行贷款项目。进一步充实招商项目储备库，夯实招商引资工作基础。

继续扩大对外贸易。落实进出口促进政策，启动“国家科技兴贸出口创新基地”建设，鼓励汽车零部件、生物化工等重点产品扩大出口，扶持有出口潜力的中小企业自营出口。坚持优势互补、共同发展原则，着力加强与沿淮城市合作，积极发展能源、煤化工产业，在建设“两淮一蚌”重化工业走廊和沿淮城市群中发挥重要作用。进一步加强与长三角、珠三角及淮海经济区的交流与合作。

    （六）加快体制机制创新，继续推动全民创业

    深化国有企业改革，加快市属企业战略性重组，积极推进华光集团等企业对外合作。加快改制企业“二次创业”，建立现代企业制度，推动顺达汽车改装厂等恢复产能。盘活存量资产，激活停产企业，促成柴油机厂等对外招商。

    进一步落实和完善全民创业政策，优化创业环境，推动全民创业。放宽创业领域，降低创业门槛，鼓励更多有创业热情、具备条件的公民创办各类企业。搭建创业平台，新建创业基地（园区）4个、创业社区4个，发挥省级创业基地的示范作用。支持创业主体顺利创业，建成市、县（区）两级创业辅导中心，为创业者提供创业咨询、开业指导、申办手续代理等方面的服务。加快创业辅导师队伍建设，大力开展创业培训。做好创业项目规划，建立创业项目库，筛选一批创业指导项目发布、推荐给创业者。化解创业瓶颈，加大对创办小企业的资金支持，积极开发和拓宽多种融资渠道，为创业者筹资融资提供条件。鼓励金融机构降低门槛，提高贷款额度，解决创业启动资金问题。力争全年个体工商户增长10%以上，私营企业增长20%以上，民营经济占国民经济的比重增加到43%以上。

    （七）加大城市建设力度，提升城市管理水平

    推进城镇化是加快经济社会发展、建设小康社会的必然要求，必须按照高起点规划、高标准建设、高效能管理、高水平经营的要求，在城镇建设和管理方面继续作出努力。今年要继续加大市区建设项目投入。改造解放路南段、燕山路东段和淮上大道，拓宽101省道南段，整治黑虎山路，进一步美化城市出口；实施淮河路、朝阳路、凤阳路、治淮路的精致化改造，完成涂山路西段的整修，重建跃进桥，提升城市主干道交通功能和景观水平；完成东海大道整修任务，建成龙子湖桥头公园，整治胜利路与解放路立交桥下的环境，加快张公山大塘综合治理和开发，完成大塘截污、清淤及部分岸线整治；完善城市基础设施，尽快开工建设黄山路、市第二污水处理厂，继续实施世行污水管网和城市排涝应急工程，加快垃圾填埋场、消防指挥中心建设，完成220千伏燕山变工程。稳步发展房地产业，改善住宅供应结构，房地产竣工面积100万平方米。争取解决市内车辆过淮河“两桥”的收费问题。

    进一步加强城市规划设计和城市管理等工作。完成12个重点区域控制性规划编制工作，编制城市综合交通体系、园林绿化、环卫设施等专业规划。深入研究发展用地问题，制定节约集约用地办法。健全城市长效管理机制，实施城市精细化管理，加大主干道和重点区域市容环境整治力度，逐步向支路和社区延伸，着力整治脏乱差现象和不文明行为，加强违法建筑防控和拆除工作，努力打造适宜人居、环境优美、整洁有序的城市新形象。

    （八）大力发展社会事业，推进和谐蚌埠建设

    发展社会事业是落实科学发展观的重要内容，构建和谐社会是我们党在新的历史时期提出的一项重大战略任务，必须认真加以落实。积极推进科技、教育、文化、医疗卫生体制改革。继续实施鼓励科技创新政策，增加科技投入，建设国家专利技术（蚌埠）展示交易中心，以多种方式推动科技成果转化。继续提高义务教育水平，调整中小学校布局，重点改造薄弱学校，扩大城乡教师交流，推进教育均衡发展；支持市二中建设新校区；大力发展职业教育，加快建设蚌埠职业技术学院（筹），积极筹建蚌埠技师学院，建成3县职教中心；实现蚌埠学院“去筹升本”目标，支持在蚌高校改善办学条件，发挥更大作用；引导民办教育健康发展。继续实施花鼓灯“三千双百”工程，对禹墟、双墩遗址进行保护性发掘。支持市第三人民医院改建和建设新院，推动医疗机构联合重组。推进社区建设，完善社区基础设施和配套服务，开展经常性社区文体活动，提高社区卫生服务覆盖率。办好市第十二届运动会，建成新体育馆主体工程和气象科普公园，完成档案馆、新博物馆选址工作。启动数字电视平移工程。

    继续加大社会保险扩面和基金征缴力度，加强监管工作，确保社保基金安全。认真执行劳动合同制和最低工资制，切实维护农民工的合法权益。积极发展慈善福利事业。严格执行建设项目环境影响评价制度，加快污染源监控中心建设，提高防控重大环境污染事件的能力。开展“五五”普法，推进依法治市。加强国防后备力量和人民防空建设，继续做好双拥共建工作。综合治理人口问题，稳定低生育水平。加强和改进信访工作，完善不稳定因素排查化解机制，继续开展领导干部开门接访、带案下访，畅通群众诉求渠道，维护群众合法权益。健全社会预警体系，加强应急管理，提高保障公共安全和处置突发事件的能力。全力做好安全生产工作，强化食品药品监管，严防重特大事故发生。进一步加强社会治安综合治理，依法严厉打击各种违法犯罪活动，推进平安蚌埠建设，让人民群众更有安全感。

    （九）高度关注民生问题，解决群众实际困难

    解决群众最关心最直接最现实的利益问题，始终是政府的重要职责。必须以群众需要为第一信号，多做实实在在的事情，努力解决涉及群众切身利益的矛盾和问题。一是努力解决群众就业难的问题。落实就业再就业优惠政策，加强劳动力培训工作，健全就业和创业服务体系，鼓励劳动者自谋职业、自主创业，全年新增城镇就业岗位3.25万个，实现下岗失业人员再就业1.6万人，其中安置“4050”人员3000人，完成农村劳动力转移培训1.1万人次。二是努力解决部分群众生活上的困难。继续完善城市居民最低生活保障制度，提高保障标准；探索建立农村最低生活保障制度；健全城乡特困群众社会救助体系。三是努力解决部分群众就医难问题。落实财政补贴资金，加快建立覆盖城乡居民的医疗保险体系；启动五河县、固镇县和淮上区新型农村合作医疗试点工作，力争参保率达到82%以上；探索建立城市困难群体大病统筹制度；加快新建17所乡镇卫生院。四是努力解决部分群众子女上学难问题。落实农村困难家庭子女上学“两免一补”政策；继续改造农村中小学危房3万平方米；重视解决城市困难家庭和进城务工人员子女就学困难。五是努力解决部分群众饮水难问题。继续实施农村安全饮水工程，使3.6万农民饮水安全得到保证。六是努力解决部分群众出行难的问题。新增市区公交线路2条、站点40个，继续改造城区支路和小街小巷27条，完成50条小街小巷路灯配套。七是努力解决部分群众居住困难。加快建设经济适用房和廉租房，建成经济适用房5万平方米以上。八是努力改善群众居住环境。加大棚户区成片旧房改造力度，做到条件成熟一个改造一个；完成4个城中村的改造；建设改造3个垃圾中转站、14座城市公厕。九是解决部分群众收听收看广播电视难的问题。年内要实现广播电视“村村通”，覆盖率达到100%。十是继续改善群众体育健身条件，完成市体育场的维修改造。在实际操作中，各级政府都要尽最大努力，争取能为群众办更多的实事。

    （十）加强政府自身建设，继续优化发展环境

    继续加强机关效能建设。坚持以发展为第一要务，破除一切不利于发展的观念、制度和做法，努力打造零障碍、低成本、高效率的区域发展环境。深化行政审批制度改革，及时清理行政审批项目和行政事业性收费项目。加强行政服务中心建设，优化工作流程，提高服务水平。规范行政执法，实施涉企处罚下限制，禁止随意到企业检查，严肃查纠行政不作为、乱作为等行为。进一步简政放权，理顺和规范部门职能。拓展政务公开内容，围绕涉及群众利益的热点问题，围绕人财物管理等容易滋生腐败的重点部位，抓住决策、执行、结果等权力运行的主要环节，公开行政权力运行全过程。坚持求真务实，转变会风、文风，提高工作效率，增强政府执行力。

    全面推行依法行政。认真贯彻《监督法》，自觉接受人大法律监督、工作监督和政协民主监督，密切与人大代表、政协委员的联系，积极支持人民政协履行职能，认真听取各民主党派、工商联和社会各界人士的意见和建议，加强与工会、共青团、妇联等人民团体的联系。认真办理人大代表议案、建议和政协提案。完善政情通报、重大事项社会公示、重大决策专家咨询等制度，提高决策的民主化、科学化水平。加快电子政务建设，继续办好政府网站、政风行风热线、政风行风面对面等沟通平台，规范新闻发布，及时公布政府工作情况。

    切实加强公务员队伍建设。加大人才培养和引进力度，进一步增强公务员的发展意识、机遇意识、忧患意识和大局观念，提高公务员素质。认真落实党风廉政建设责任制，强化行政监察和审计监督职能，预防和治理腐败。加强政务督查，完善目标考核，继续在政府部门开展述职评议和评优评差活动，实行行政问责和末位惩诫。增强政府工作人员责任心，努力建设责任政府。

    各位代表！加快蚌埠发展的任务艰巨，责任重大，前景光明。让我们在省委、省政府和中共蚌埠市委的坚强领导下，高举邓小平理论和“三个代表”重要思想伟大旗帜，全面落实科学发展观，凝心聚力，团结拼搏，为圆满完成今年各项任务，为实现蚌埠早日崛起和人民安康富裕而继续努力奋斗！
